# A Remarkable Calculation

**Published:** 1892-06

**Authors:** 


### A REMARKABLE CALCULATION.

Canon Sydney Smith, equally celebrated as a *bon vivant* and a wit, at the termination of his life, writes thus to his friend, Lord Murray : You are, I hear, attending more to diet than heretofore. If you wish for any thing like happiness in the fifth act of life, eat and drink only about one-half what you could eat and drink. Did I ever tell you my calculation about eating and drinking ? Having ascertained the weight of what I could live upon so as to preserve health and strength, and what I did live upon, I found that, between ten and seventy years of age, I had eaten and drunk forty-four wagon-loads of meat and drink more than would have preserved me in life and health ! The value of this mass of nourishment I consider to be worth seven thousand pounds sterling. It occurred to me that I must have starved to death fully a hundred persons. This is a frightful calculation, but irresistibly true.

